# A novel technique of quantifying first metatarsophalangeal (1^st^ MPJ) joint stiffness

**DOI:** 10.1186/1757-1146-7-S1-A32

**Published:** 2014-04-08

**Authors:** Marabelle L Heng, Pui W Kong

**Affiliations:** 1 Physical Education & Sports Science Academic Group, National Institute of Education, Nanyang Technological University, Singapore 637616; 2 Podiatry Department, Singapore General Hospital, Singapore 169608

## 

**Figure 1:**
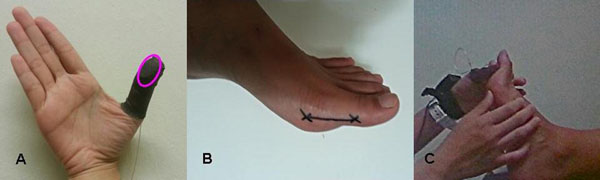
**A.** Finger sleeve with pressure pad (circled) on tip of thumb to measure force applied to move the 1^st^ MPJ. **B.** Moment arm (length of proximal phalanx) from joint fulcrum to point of force application. **C.** Displacement force applied to proximal phalanx, dorsiflexing 1^st^ MPJ through its range of motion.

The first metatarsophalangeal joint (1^st^ MPJ) mobility is usually described by (i) range of motion in degrees (°) or (ii) stiffness based on an experienced tester’s subjective feel, ie. hypermobile, normal or stiff. Approximately 65° of 1^st^ MPJ dorsiflexion is required for normal effective walking [1]. Visual estimation of 1^st^ MPJ range of motion is often used in current practice [2], reflecting the absence of a reliable and practical method for clinicians to quantify 1^st^ MPJ stiffness. This study presents a novel technique to measure joint stiffness using a tactile pressure sensing system (Figure 1A) together with simple video analysis.

**Figure 2:**
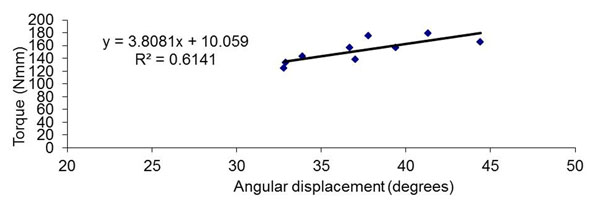
Plot of torque (Nmm) against angular displacement (degrees) of the 1^st^ MPJ to determine joint stiffness from the slope.

To illustrate the method, data were collected on one female flat-footed subject with posterior tibial tendon dysfunction (age 25 yr, body mass index 20.6 kg/m^2^). The moment arm was measured from the tuberosity of the first metatarsal head to just beneath the tuberosity of the 1^st^ distal phalanx (Figure 1B). A qualified podiatrist moved the 1^st^ MPJ of the subject through its full range of motion before data collection. For each trial, joint movement is paused briefly at 3 interval points between the resting and maximally dorsiflexed position. At each interval point, the corresponding force applied was measured using a tactile pressure sensing system (Figure 1C). The procedures were recorded by a synchronised webcam such that the angular displacement of the 1^st^ MPJ can be quantified using video analysis. A total of 3 trials were taken, resulting in nine sets of data points to plot a torque-angular displacement graph (Figure 2). The joint stiffness was then calculated as the slope of the line of best fit as 3.8 Nmm/deg. The R^2^ indicates that 61% of variability can be explained by this model.

The proposed method of quantifying 1^st^ MPJ stiffness is potentially useful for measuring small joint stiffness in clinical practice. Quantified joint stiffness provides greater accuracy to facilitate clinicians in their diagnoses and prescription of treatment.
